# Salerno—Naples

**Published:** 1860-07

**Authors:** Wm. Clendenin


[*Cincinnati Lancet and Obs.*

### ARTICLE XIV.

### *Salerno—Naples.*

Salerno was in the twelfth century, the seat of a flourishing medical college—the first, perhaps, established west of Greece. According to Gibbon, "The treasures of Grecian medicine had been communicated to the Arabian Colonies of Africa, Spain, and Sicily; and in the intercourse of peace and war a spark of knowledge had been kindled and cherished at Salerno, an illustrious city in which the men were honest and the women beautiful." I made a pilgrimage to this "illustrious" city, which, on account of its associations, I enjoyed very much. Yet, sad to relate, I could find neither "honest men nor beautiful women," and the *only* remains of the medical school that could discover was a "string of aphorisms in Leonine verses of the twelfth century." These aphorisms were the maxims of the School of Salerno. This precious relic is now in the library of the Benedictine Monastery, which is situated high up amongst the Apennines, about five miles from Salerno. I copied from these aphorisms the following eulogium of the virtues of sage tea:

> "Cur moriatur homo, cui salvia crescit in horto?
> Contra vim mortis non est medicamen in hortis?
> Salvia salvatrix, naturæ conciliatrix,
> Salvia cum ruta faciunt tibi pocula tuta."

I dare say you have seen this eulogium before; but I send it as copied *literatim et*, etc., from the "string."

The following, also from the same "string," is of a different character, and I have no doubt was more commonly used than the sage tea:

> "Si nocturna tibi noceat potatio vini
> Hoc ter mane bibes iterum, et fuerit medicina."

The following "rules and regulations" of the School of Salerno were kindly furnished me by Padre Rossi, the archivist of the above named monastery. No person was allowed to practice medicine in the kingdom who had not been examined by this college. Proofs of legitimacy, and of having studied medicine for seven years, were required from the candidates. The examinations were public, and consisted of expositions of Galen, Hippocrates, Avicenna, etc. After a satisfactory examination, the student was to practice one year under a physician. Druggists were not allowed to dispense medicines until they had received a certificate of capability from the college.

Salerno is now a poor, deserted-looking old city. The physicians of the town seem to be "practicing medicine" and living entirely upon the reputation of their ancestors.

The medical school at Naples seems to be in a flourishing condition. The number of pupils at present is one hundred and sixteen. The students of this school all wear a uniform of blue cloth, and a hat, which, in form and color, resembles that worn by our old Continental officers. A student entering this college is obliged to remain seven years. Clinical lectures are delivered daily, in the morning, at the Hospital Degl. Incurabili.

Perhaps no city in Europe is better supplied with hospitals than Naples. The revenues, which are very large, are administered by a president and three governors appointed by the king. The "Incurabili" is a vast establishment for both sexes, having separate wards for particular diseases. The average number of patients in this hospital is two thousand. Each hospital has a convalescent establishment in the country, to which convalescents are sent. Patients whose cases are considered hopeless are sent to the "dying ward"—a barbarous and inhuman practice.

Tuberculosis is considered contagious. The Albergo de Poveri is a vast building founded by Charles III., as an asylum where all the poor of the kingdom might be received and taught some useful occupation. It contains at present about 5,500 inmates. The boys brought up in this institution are usually sent into the army. The hospital for the blind contains about two hundred inmates; it is well conducted. Besides these, there are seven other large hospitals in Naples; also, various private charitable institutions.

I think the general plan and arrangement of the hospitals of Rome superior to any I have seen in Europe. In these hospitals the wards converge to a centre where the altar stands under a dome. This form is certainly well calculated to contribute to good ventilation, and renders the service easier and more economical.

From your friend, WM. CLENDENIN.

